# Supplementary material for: Specification of diverse cell types during early neurogenesis of the mouse cerebellum
Source: eLife. 2019 Feb 8;8:e42388. doi: 10.7554/eLife.42388 (PMC6382353; doi:10.7554/eLife.42388)
Supplement: Supplementary file 3. — Lists of primary and secondary antibodies used in the current study. [file elife-42388-supp3.docx]

**Supplementary file 3. List of antibodies used in the current study**

| **Antigen** | **Symbol** | **Species** | **Dilution** | **Vendor** | **Cat. #** | **RRID** |
| --- | --- | --- | --- | --- | --- | --- |
| CALBINDIN, 28KD | Calb1 | Rabbit | 1:3000 | Swant | CB-38a | AB-2721225 |
| CALRETININ | Calb2 | Rabbit | 1:1000 | Swant | 7699/4 | AB_2313763 |
| Dab1 | Dab1 | Mouse | 1:200 | Santa Cruz | sc-271136 | AB_10610240 |
| Ebf1-4 | Ebf | Mouse | 1:1000 | Santa Cruz | sc137065 | AB_2246405 |
| FGF17 | Fgf17 | Mouse | 1:200 | Santa Cruz | sc-376056 | AB_1089091 |
| FOXP1 | Foxp1 | Mouse | 1:400 | Santa Cruz | sc-398811 |  |
| FOXP1 | Foxp1 | Guinea pig | 1:500 | Gift from Dr. Novitch |  |  |
| FOXP2 | Foxp2 | Goat | 1:2000 | Everest Biotech | EB05226 | AB_2107112 |
| ISL1 | Isl1 | Mouse | 1:100 | DSH Bank | 39.4D5 | AB_2314683 |
| LMX1A | Lmx1a | Rabbit | 1:1000 | Millipore | AB10533 | AB_10805970 |
| MEIS2 | Meis2 | Mouse | 1:200 | Santa Cruz | sc-515470 |  |
| NEUROGRANIN | Nrgn | Rabbit | 1:750 | Millipore | AB5620 | AB_91937 |
| OTX2 | Otx2 | Goat | 1:400 | R&D Systems | AF1979 | AB_2157172 |
| pERK1/2 | pERK | Rabbit | 1:1000 | Cell signaling | #4370 | AB_2315112 |
| SOX2 | Sox2 | Goat | 1:250 | R&D Systems | AF2018 | AB-355110 |
| TFAP2B | Tfap2b | Mouse | 1:200 | Santa Cruz | sc-390119 |  |
| Wls | Wls | Rabbit | 1:2000 | Seven Hill Bioreagents | RLAB-177 |  |
